# Effect of Pneumococcal Conjugate Vaccination in Uruguay, a Middle-Income Country

**DOI:** 10.1371/journal.pone.0112337

**Published:** 2014-11-06

**Authors:** Gabriela García Gabarrot, Mariana López Vega, Gabriel Pérez Giffoni, Silvia Hernández, Pablo Cardinal, Viviana Félix, Jean Marc Gabastou, Teresa Camou, the Uruguayan SIREVA II Group

**Affiliations:** 1 Departamento de Laboratorios, Ministerio de Salud Pública, Montevideo, Uruguay; 2 Unidad de Terapia Intensiva, CASMU-IAMPP, Montevideo, Uruguay; 3 Area of Technology and Health Services Delivery, Pan American Health Organization, Washington, D. C., United States of America; 4 Red de Laboratorios, Ministerio de Salud Pública, Montevideo, Uruguay; Centers for Disease Control & Prevention, United States of America

## Abstract

**Background:**

In 2008, a 7-valent pneumococcal conjugate vaccine (PCV7) was introduced into the routine childhood immunization program in Uruguay, with a 2+1 schedule. In 2010, PCV13 replaced PCV7, and the same 2+1 schedule was used. The effect of these pneumococcal vaccines on the incidence of invasive pneumococcal infections (IPD) and on serotype distribution was analyzed retrospectively, based on passive national laboratory surveillance.

**Methods:**

Data from 1,887 IPD isolates from 5 years before and 5 years after PCV7 introduction (7 before and 3 after PCV13 introduction) was examined to assess the incidence rate per 100,000 age-specific population of all IPD, PCV7-serotypes, and PCV13-serotypes associated IPD among children <2 years and 2 to 4 years old, and patients ≥5 years old. Trends of frequency for each serotype were also analyzed.

**Results:**

Comparison of pre-vaccination (2003–2007) and post-vaccination (2008–2012) periods showed a significant decrease in IPD incidence among children <2 years old (IR 68.7 to IR 29.6, p<0.001) and children 2 to 4 years (p<0.04). IPD caused by serotypes in PCV7 was reduced by 95.6% and IPD caused by 6 serotypes added in PCV13 was reduced by 83.9% in children <5 years old. Indirect effects of both conjugate vaccines were observed among patients ≥5 years old one year after the introduction of each vaccine, in 2010 for PCV7 and in 2012 for PCV13. Nevertheless, for reasons that still need to be explained, perhaps due to ascertainment bias, total IPD in this group increased after 2007. In 2012, the relative frequency of vaccine serotypes among vaccinated and unvaccinated population declined, except for serotype 3. Non vaccine serotypes with increasing frequency were identified, in rank order: 12F, 8, 24F, 22F, 24A, 15C, 9N, 10A and 33.

**Conclusion:**

Consecutive immunization with PCV7 and PCV13 has significantly reduced IPD in children <5 years of age in Uruguay.

## Introduction

Invasive pneumococcal disease (IPD) is still a major cause of morbidity and mortality in children under 5 years old, the elderly and immunocompromised patients. The epidemiology of pneumococcal infections has begun to change following the introduction of conjugate vaccines.

A seven-valent pneumococcal conjugate vaccine (PCV7), first introduced in the United States in 2000, has been remarkably effective in reducing IPD caused by pneumococci of serotypes included in the formula (4, 6B, 9V, 14, 18C, 19F and 23F) among vaccinated children. An indirect or “herd” effect on IPD caused by PCV7 vaccine-types (VT-PCV7) was also observed in unvaccinated groups, as a result of reduced carriage and transmission of these serotypes [1].

However, while VT-PCV7 virtually disappeared as a cause of IPD in countries with >3 years vaccination, serotype replacement and emergence of “new” serotypes have been observed, first as an increase in carriage and later as an increase in invasive disease [2], [3]. Vaccine-escape recombinants, for instance a serotype 19A strain, emerged by capsular switch of a serotype 4 strain and expanded in United States and Canada [4], [5].

In 2010, a 10-valent pneumococcal conjugate vaccine (with additional serotypes 1, 5 and 7F) and a 13-valent pneumococcal conjugate vaccine (PCV13, with additional serotypes 1, 3, 5, 6A, 7F and 19A) became available for implementation into national immunization programs. The serotypes included in the new conjugate vaccines offered better potential coverage to countries where VT-PCV7 were less represented among IPD isolates, such as Latin American countries [6]. Before the introduction of PCV7, the lowest potential coverage among Latin American countries was recorded in Uruguay (1994–2001), where VT-PCV7 represented 53% of IPD among children <2 years old and 41% among children 2 to 5 years old [7].

Hopefully, extended formulations will prevent most IPD among children <5 years globally, but concern remains about the extent to which replacement by non vaccine types (NVT) or capsular switching might undermine the impact of the new extended valency vaccines in the long-term. Published studies suggest that the magnitude of replacement disease is lower than the magnitude of decline in vaccine types-related disease in most countries where routine pneumococcal conjugate vaccination has been introduced [8]. However, most published studies have been from industrialized countries with different social and economic situations from those of low-and middle-income countries, also from countries with different serotype distributions.

We present a retrospective analysis of the effects of PCV7- and PCV13- vaccination in Uruguay. Differences in the IPD incidence caused by VT-PCV7, the 6 additional serotypes in PCV13 (VT-PCV13) and NVT were estimated among the vaccinated and unvaccinated population, 5 years before and 5 years after the introduction of conjugate vaccines (2003–2012). We have also analyzed the relative frequency changes for each vaccine-serotype and for emerging non vaccine serotypes.

## Methods

A retrospective, population-based cohort study, based on passive national laboratory surveillance of IPD, was performed.

As our laboratory is the National Public Health Reference Center for *S. pneumoniae* surveillance, we regularly receive isolates with enclosed relevant patient information. Our routine protocol assigns a laboratory number to identify each isolate. After that the patient records/information is anonymized and de-identified prior to analysis. This retrospective study has been approved by Dirección General de Salud, Ministerio de Salud Pública.

Uruguay is a middle-income country, one of the smallest in South America, with a population of 3.3 million and only 240,000 children aged <5 years [9].

In March 2008, PCV7 was introduced into the routine childhood immunization program in Uruguay, with a 2+1 schedule (doses administered at 2, 4 and 12 months of age). During the first year, a 2 dose catch-up program was also offered to children up to 2 years old. High vaccine coverage was achieved and effectiveness reached 94% [10]. In March 2010, PCV13 replaced PCV7, and the same 2+1 schedule was used. Children with 1 or 2 doses of PCV7 completed their schedule with PCV13. Additionally, between April and August 2010, a single dose catch-up of PCV13 was offered to all children born between 2005 and 2008. Vaccine coverage among new cohorts has been 96.9–98.9% for the first dose and 91.3–93.2% for the complete schedule [11].

Since 1999, the polysaccharide 23-valent pneumococcal vaccine (PPSV23) (serotypes 1, 2, 3, 4, 5, 6B, 7F, 8, 9N, 9V, 10A, 11A, 12F, 14, 15B, 17F, 18C, 19F, 19A, 20, 22F, 23F and 33F) has been offered through the Public Health system to patients >5 years old with increased risk for IPD and also to adults ≥65 years old, but it is presumed to have low coverage among the target population. During 2011–2012 only 40,000 doses were provided to the target population by the Ministry of Health (Teresa Picón, personal communication).

### National surveillance of IPD

Laboratory-based surveillance of IPD started in 1987 [12] and became nationwide in 1994, when a regional pneumococcal network called SIREVA, was organized. The network, aimed at monitoring IPD serotypes distribution, was coordinated by the Pan American Health Organization (PAHO), and 6 countries, including Uruguay, were invited to participate [6], [13]. Consensus procedures and external quality assurance control were established and have been described elsewhere [14]. Later the network was expanded to include almost all Latin American and Caribbean countries [15].

In Uruguay, passive laboratory-based surveillance is not mandatory. IPD isolates from public and private institutions all over the country are sent by clinical microbiologists to Departamento de Laboratorios, the National Public Health Reference Laboratory.

Bacterial identification was confirmed, and serotyping was performed by Quellung with pools and specific antisera from Statens Serum Institut, Denmark. Sera to identify the 13 serotypes in PCV13 were available from the beginning of the study, and the number of other serotypes we are able to identify has increased over time with the acquisition of new typing sera which have been utilized retrospectively. Serotype 6C was identified by PCR until specific serum was commercially available [16].

### Analysis of data

Laboratory records were examined searching for serotypes of all IPD cases between January 2003 and December 2012. Incidence per 100,000 population of total cases of IPD, IPD caused by VT-PCV7, IPD caused by VT-PCV13 and IPD caused by NVT, was calculated for children <2 years, 2–4 years and 5–14 years old. The same analysis was performed for adults 15–59 years and ≥60 years old.

Changes in incidence rates (IR) were presented as incidence rate ratio with 95% confidence intervals (CI) and percent changes. Proportions of pneumococcal isolates by clinical diagnosis were tested with Chi-square test or Fisher exact test, as required. A p<0.05 was considered to be significant. Statistical analysis was performed with Epi Info, version 3.5.4 [17].

The year 2008, when PCV7 was introduced in the national immunization program, was considered a transition year and was not included in statistical analysis, except for incidence trends throughout the whole period.

## Results

Between 2003 and 2012, 1,887 invasive pneumococcal isolates were identified: 478 isolates were from children <2 years old, 264 isolates were from children 2–4 years old, and 1145 were from patients ≥5 years old. For incidence rates (IR) calculation, the last group was further subdivided into 3 groups: 5–14 years old (n = 207), 15–59 years old (n = 499) and ≥60 years old (n = 439).

Pneumonia was the most frequent clinical diagnosis (n = 1450, 78%), followed by meningitis (n = 247, 13%), bacteremia/sepsis (n = 115, 6%) and other invasive diseases (n = 54, 3%). Significant changes in proportion for each clinical diagnosis were not observed comparing 2 periods, 2003–2007 and 2009–2012 (p>0.05) (Table 1).

**Table 1:** Invasive pneumococcal disease by age and clinical diagnosis.

Patients <2 years	2003–2007			2009–2012		
n = 422	N	Average	%	N	Average	%
Pneumonia	227	45.4	68,2	62	15.5	69.7
Meningitis	51	10.2	15.3	16	4	17.9
Bacteremia/sepsis	44	8.8	13.2	8	2	9.0
Other IPD	11	2.2	3,3	3	0.75	3.4
Total	333	66.6	100.0	89	22.25	100.0
ND	3			0		

Patients 2–4 years	2003–2007			2009–2012		
n = 241	N	Average	%	N	Average	%
Pneumonia	163	32.6	91,6	59	14.8	93.6
Meningitis	6	1.2	3,4	2	0.5	3.2
Bacteremia/sepsis	6	1.2	3,4	2	0.5	3.2
Other IPD	3	0.6	1,6	0	0	0
Total	178	35.6	100.0	63	15.8	100.0
ND	2			0		

Patients ≥5 years	2003–2007			2009–2012		
n = 1019	N	Average	%	n	Average	%
Pneumonia	292	58.4	74,9	480	120	78.0
Meningitis	66	13.2	16.9	88	22	14.3
Bacteremia/sepsis	18	3.6	4.6	31	7.8	5.1
Other IPD	14	2.8	3,6	16	4	2.6
Total	390	78	100.0	615	153.8	100.0
ND	7			7		

ND: No data about clinical diagnosis; Other IPD: peritonitis, cellulitis, septic arthritis, osteomyelitis, abscesses.

### Incidence of IPD

**Figure 1:**
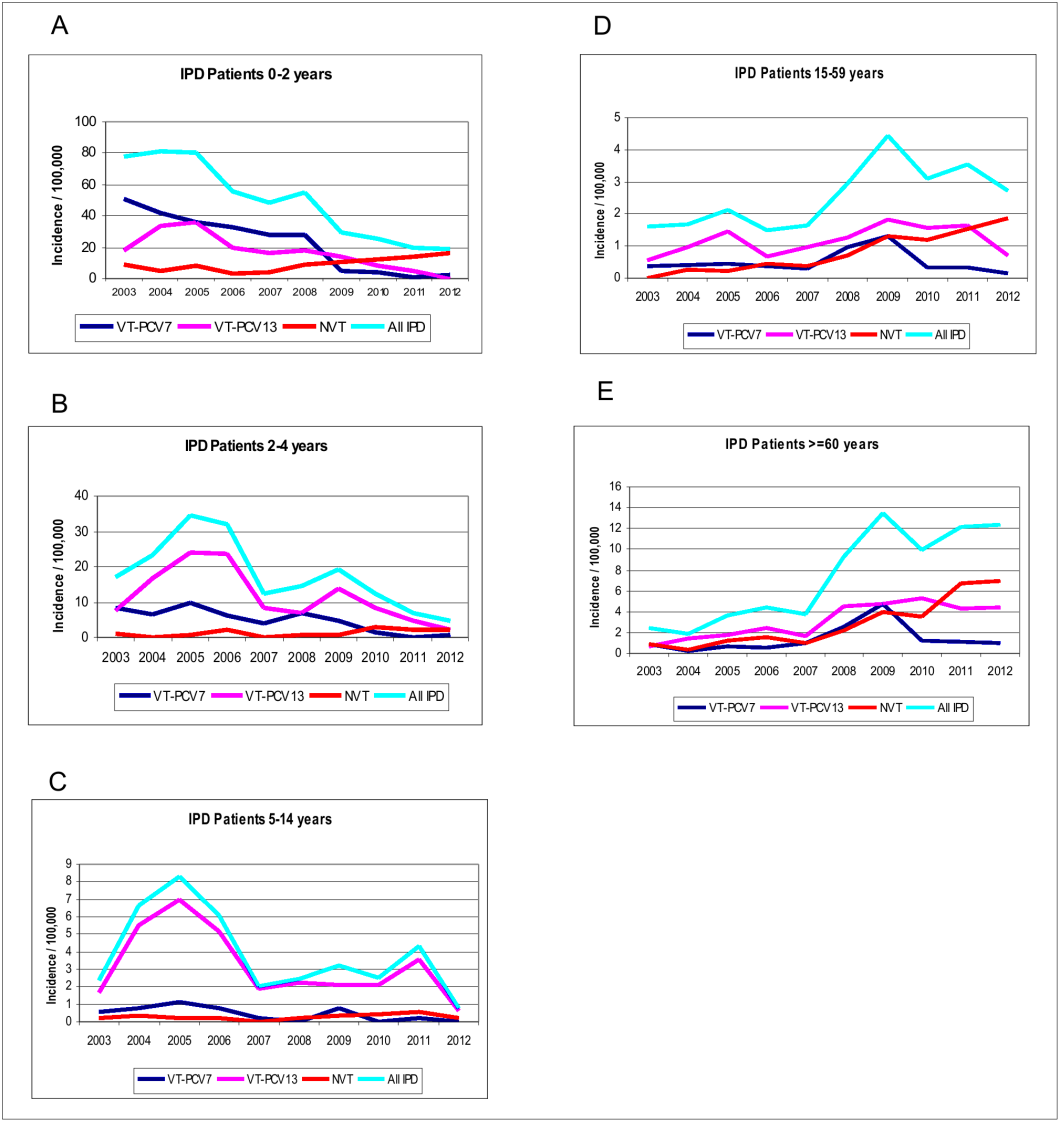
Incidence of invasive pneumococcal disease (IPD) per 100,000 population: A) patients 0–2 years old; B) patients 2–4 years old; C) patients 5–14 years old; D) patients 15–59 years old; E) patients ≥60 years old. VT-PCV7: IPD caused by PCV7 serotypes; VT-PCV13: IPD caused by 6 additional serotypes in PCV13; NVT: non vaccine serotypes.

The highest IR of IPD among children <2 years old before the introduction of conjugate vaccines, was 80/100,000 in 2005, while it reached 19/100,000 in 2011 (Figure 1A). A significant reduction of IR between pre- (2003–2007) and post- (2008–2012) vaccination period, was observed in this group of age, from 68.7 to 23.25 for total IPD cases (Table 2). Differences between both periods were also significant for the same age group when VT-PCV7 and for VT-PCV13 associated IPD was analyzed. An increase in incidence of IPD caused by NVT was also observed, though this was not statistically significant (p = 0.0694) (Table 2). The greatest incidence of VT-PCV13 associated disease was observed in 2005 (36/100,000) due to the high incidence of IPD caused by serotypes 1 and 5 (data not shown). Similar peaks were also observed in older children and adults <60 years old (Figure 1B, 1C, 1D).

**Table 2:** Incidence rates ratios of IPD between pre- and post-vaccination periods by age group.

	2003–2007		2009–2012		IRR (95%CI)			p value
	Cases	IR	Cases	IR				
**<2 years**								
All IPD	336	68.68	89	23.25	0.34	0.211–0.543		0.0001
VT-PCV7	186	37.99	12	3.13	0.08	0.025–0.263		0.0001
VT-PCV13	121	24.78	26	6.79	0.25	0.102–0.612		0.0024
NVT	29	5.92	51	13.33	2.60	0.927–7.293		0.0694
Population	97,845		95,699					
**2–4 years**								
All IPD	180	23.82	63	10.93	0.43	0.207–0.913		0.0279
VT-PCV7	53	6.97	10	1.73	0.17	0.020–0.138		0.0971
VT-PCV13	121	16.05	42	7.29	0.44	0.180–1.063		0.0681
NVT	6	0.79	11	1.92	3.00	0.122–73.647		0.5011
Population	151,133		144,099					
**5–14 years**								
All IPD	138	5.08	56	2.75	5.00	0.240–104.150		0.2989
VT-PCV7	18	0.66	5	0.24	0.09	0.005–1.644		0.1045
VT-PCV13	115	4.24	43	2.09	5.00	0.240–104.151		0.2989
NVT	5	0.18	8	0.38	9.50	3.384–26.607		0.001
Population	543,307		513,510					
**15–59 years**								
All IPD	165	1.70	276	3.45	3.00	0.312–28.841		0.3414
VT-PCV7	37	0.38	43	0.54	1.00	0.020–50.400		1
VT-PCV13	90	0.93	114	1.42	3.00	0.122–73.647		0.5011
NVT	38	0.39	119	1.48	3.00	0.122–73.647		0.5011
Population	1,941,176		2,010135					
**≥60 years**								
All IPD	94	3.23	290	11.98	3.67	1.023–13.143		0.0461
VT-PCV7	19	0.65	48	1.99	3.00	0.122–73.646		0.5011
VT-PCV13	46	1.58	113	4.67	4.00	0.447–35.788		0.215
NVT	29	0.99	129	5.31	11.00	0.608–198.938		0.1045
Population	582,043		605,175					

IR: incidence rate; IRR: incidence rate ratio; CI: confidence interval.

Significant differences (p<0.05).

For children 2–4 years old, the incidence of IPD also peaked in 2005 with 35/100,000 population and another in 2009 with 19/100,000, also due to increased frequency of serotypes 1 and 5 (data not shown). Declines to 7/100,000 and 5/100,000 were observed in 2011 and 2012 (Figure 1B). Among this group, the incidence rates ratio between pre- and post-vaccination periods was significant for IPD caused by any serotype, but it did not reach statistical significance for VT-PCV7, VT-PCV13 and NVT associated IPD (Table 2).

Temporal trends of IPD incidence among older children (5–14 years) were similar to those observed for children 2–4 years, with peaks in 2005 and 2009, but also in 2011. Among this unvaccinated population the 2009 peak was due to increase in VT-PCV7 IPD. The same situation was observed for patients 15–59 years old and for patients ≥60 years old (Figure 1C, 1D, 1E). Among patients 5–14 years old, IR varied between 2 and 8/100,000 during the 2003–2007 period and declined to 0.7/100,000 in 2012 (Figure 1C).

An increase in the incidence of IPD among patients ≥15 years was observed after PCV7 introduction. For patients 15–59 years old, IR was 1.6 in 2003 and reached 4.4 in 2009. Among patients ≥60 years, differences in IR before and after vaccination were larger, from 2.4 in 2003 to 13.4 in 2009.

VT-PCV7 associated IPD incidence remained below 1/100,000 for patients 15–59 years and patients ≥60 years during the pre-vaccination period. In 2009, a peak was observed among both age groups in IPD caused by any serotype and IPD caused by VT-PCV7. The IR of VT-PCV13 associated IPD declined since 2011 for patients 15–59 years old but not for patients ≥60 years old (Figure 1D, 1E).

Increases in IPD caused by NVT were observed at all ages and approached a significant p value among children <2 years old (p = 0.0694). Incidence rates ratios were not statistically significant among older patients (p>0.05; Table 2).

### Trends of IPD caused by PCV7 and PCV13 serotypes

Trends of IPD caused by VT-PCV7 and/or VT-PCV13 were compared between three periods: 2003–2007, 2009–2010 and 2011–2012, for patients <5, 5–14, 15–59 and ≥60 years old (Table 3).

**Table 3:** Trends of IPD caused by VT-PCV7, VT-PCV13 and other serotypes.

	2003–2007		2009–2010		2011–2012		Change
	n	IR	n	IR	N	IR	%
**<5 years old**	516		98		54		
VT-PCV7	239	19.10	18	3.75	4	0.84	−95.6
VT-PCV13	242	19.45	53	11.04	15	3.14	−83.9
VT-PCV7+VT-PCV13	481	38.55	71	14.79	19	3.98	−89.7
NVT	35	2.79	27	5.63	35	7.33	162.7
**5–14 years old**	138		30		26		
VT-PCV7	18	0.66	4	0.38	1	0.10	−84.8
VT-PCV13	115	4.24	22	2.12	21	2.07	−51.2
VT-PCV7+VT-PCV13	133	4.90	26	2.50	22	2.17	−55.7
NVT	5	0.18	4	0.39	4	0.40	122.2
**15–59 years old**	165		150		126		
VT-PCV7	37	0.38	33	0.83	10	0.25	−34.2
VT-PCV13	90	0.93	67	1.68	47	1.17	25.8
VT-PCV7+VT-PCV13	127	1.31	100	2.51	57	1.42	8.4
NVT	38	0.39	50	1.26	69	1.71	338.4
**> = 60 years old**	94		140		150		
VT-PCV7	19	0.65	35	2.93	14	1.06	63.1
VT-PCV13	46	1.58	60	5.01	52	4.33	174.1
VT-PCV7+VT-PCV13	65	2.23	95	7.94	66	5.39	141.7
NVT	29	0.99	45	3.76	84	6.87	593.9

VT-PCV7 IPD was reduced by 95.6% among children <5 years old and varied between 34.2% and 84.8% among patients ≥5 years old. VT-PCV13 IPD was reduced by 83.9% among children <5 years old and varied between 25.8% and 174.1% among patients ≥5 years old. NVT related IPD increased along the 3 periods among all age groups, especially among the unvaccinated population (Table 3).

**Figure 2:**
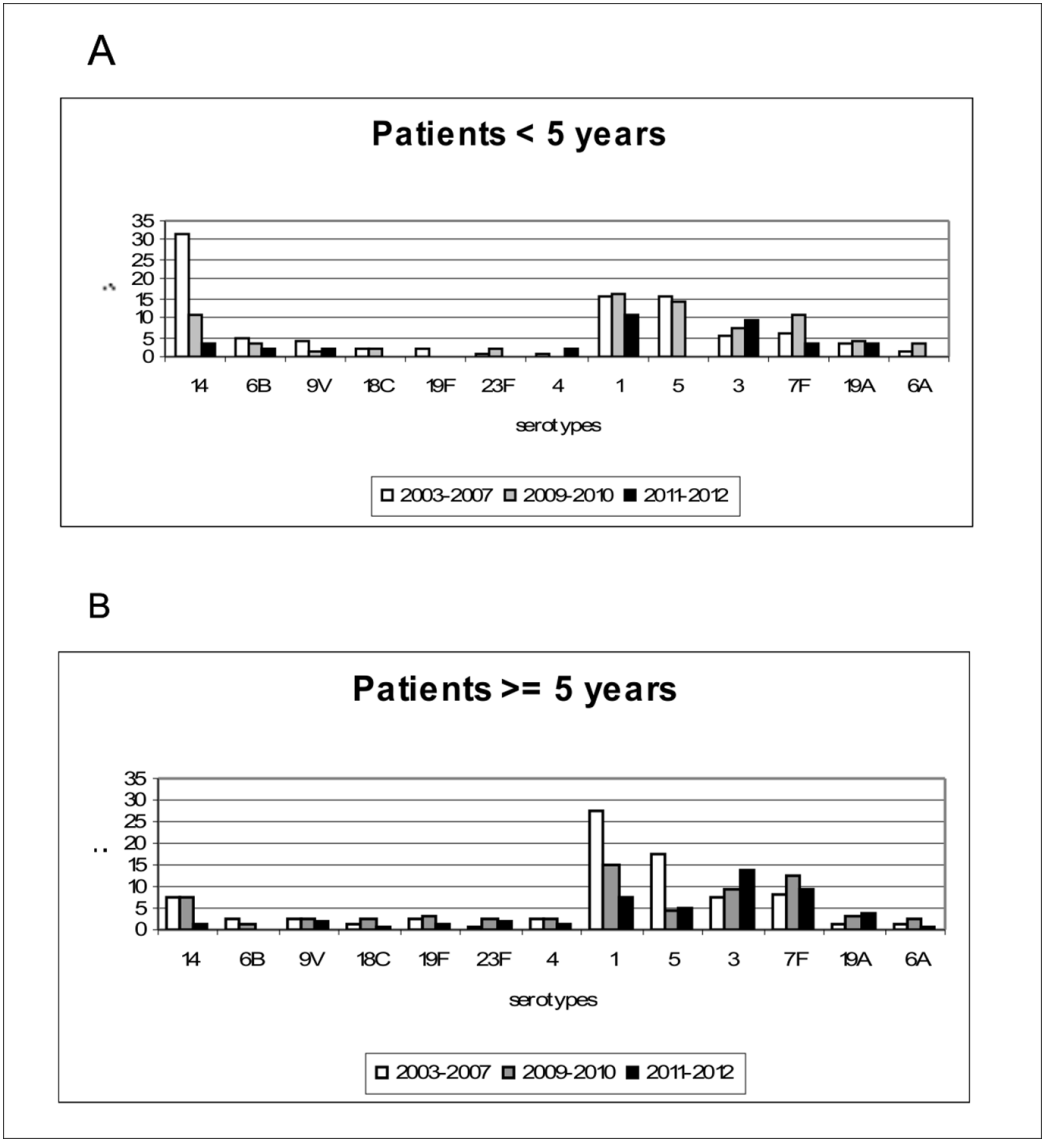
Relative frequency of 13 conjugate vaccine serotypes before and after PCV7 and PCV13 introduction: A) patients <5 years old; B) patients ≥5 years old. PCV7 and PCV13 were introduced into national immunization program in 2008 and 2010, respectively.

Comparisons of relative frequencies for each vaccine serotype were carried out for the same periods among 2 age groups: <5 and ≥5 years old (Figure 2). Among VT-PCV7, the most important reduction was observed for serotype 14, the predominant serotype among children <5 years old before vaccination.

VT-PCV13 isolates, except for serotype 5, increased in 2009–2010 among children <5 years old, and isolates of serotypes 3, 6A, 7F and 19A also increased among the non vaccinated population. In 2011–2012 a decrease in VT-PCV13 was observed among all patients except for serotype 3. Serotype 3 increased during the two periods post-vaccination, within both the vaccinated and non vaccinated population (Figure 2).

The percentage of IPD isolates of serotypes included in PPSV23 among patients ≥5 years old was 92.7% during the first period, and declined to 86.3% and 86.4% during the second and third periods (data not shown).

### Meningitis

A reduction of 59% in the number of cases of pneumococcal meningitis in children <2 years old was observed, from an average of 10.2 cases per year during 2003–2007, to 4 cases per year during 2009–2012 (p<0.05) (Table 1). During the pre-vaccination period, 19 children within this group died of pneumococcal meningitis (case fatality rate 37.3%), while 6 died during the post-vaccination period (case fatality rate 28.6%). Among this age group, there was a decrease in VT-PCV7 from 21 to 5 isolates comparing pre- and post-vaccination period. VT-PCV13 represented 45% of the cases of meningitis between 2003 and 2009 (28/62), but only 1 out of 3 cases of meningitis in 2010 and none in 2011–2012.

In contrast, an increase of 45% in pneumococcal meningitis cases was seen among patients ≥5 years old, from 13.2 cases per year during 2003–2007 to 22 cases per year during 2009–2012 (Table 1). Twenty- two patients in this group died during the pre-vaccination period (case fatality rate 33.3%), while 27 died during the post-vaccination period (case fatality rate 28.1%).

The relative frequency of VT-PCV7 related meningitis among patients ≥5 years old decreased after vaccination from 22.7% (15/66) to 14.6% (14/96), while VT-PCV13 represented 23.9% of meningitis cases in this age group between 2003 and 2009 (21/88) and 17.6% during 2010–2012 (13/74).

### Emerging non vaccine serotypes

Absolute and relative frequencies of all NVT IPD isolates among children <5 years were calculated: 9 NVT showed an increased frequency comparing pre- and post- vaccination periods in this age group, and most of these serotypes also increased among patients ≥5 years old (Table 4).

**Table 4:** Non-vaccine serotypes with increased frequency at post-vaccination period.

Serotypes	<5 years	≥5 years		Total
	2003–2007	2008–2012	2003–2007	2008–2012	P	2003–2007	2008–2012
	n (%)	n (%)	n (%)	n (%)		N (%)	n (%)
12F	3 (0.6)	11 (4.9)	17 (4.3)	83 (11.1)	0.0001	20 (2.2)	94 (9.7)
8	2 (0.4)	3 (1.3)	11 (2.8)	42 (5.6)	0,0292	13 (1.4)	45 (4.6)
24F	6 (1.2)	12 (5.3)	1 (0.3)	8 (1.1)	0,00179	7 (0.8)	20 (2.1)
22F	1 (0.2)	4 (1.8)	5 (1.2)	26 (3.5)	0,0278	6 (0.7)	30 (3.1)
24A	0	5 (2.2)	0	2 (0.3)	0,546	0	7 (0.7)
15C	0	3 (1.3)	1 (0.3)	3 (0.4)	1	1 (0.1)	6 (0.6)
9N	1 (0.2)	3 (1.3)	1 (0.3)	9 (1.2)	0,1785	2 (0.2)	12 (1.2)
10A	0	3 (1.3)	2 (0.5)	4 (0.5)	1	2 (0.2)	7 (0.7)
33*	0	4 (1.8)	2 (0.5)	1 (0.1)	0,2771	2 (0.2)	5 (0.5)
Total IPD	516 (100)	226 (100)	397 (100)	748 (100)		913 (100)	974 (100)

The most frequent NVT among children <5 years old before the introduction of conjugate vaccines was serotype 24F. Although it only caused 1.2% of IPD, the number of isolates increased from 6 to 12 after vaccination (p<0.001). The most frequent NVT among ≥5 years old before vaccination was serotype 12F, with 4.3% of isolates. A significant increase in the number of isolates of this serotype at all ages was observed in the post-vaccination period (p<0.001). Moreover, during the last five years, serotypes 24F and 12F ranked first and second among children <5 years of age. Other emerging NVT with significant increases in the number of isolates were 8 and 22F (p<0.05), which increased 5-fold. Other NVT with increased number of isolates were 24A, 15C, 9N, 10A and 33* although these changes were not statistically significant (Table 4).

## Discussion

The aim of this study was to evaluate the effect of pneumococcal conjugate vaccination in Uruguay, a middle-income country. The results demonstrated a significant reduction of IPD in children <5 years of age after consecutive immunization with PCV7 and PCV13. Among the most representative group, children under 2 years of age, IPD incidence rates dropped from 68.7 to 23.25 between pre- (2003–2007) and post- (2008–2012) vaccination periods.

Before PCV7 vaccination, the IPD incidence rate was 68.7/100,000 for Uruguayan children <2 years old, compared with 44.4/100,000 reported for Europe and 60/100,000 for South Africa before the HIV epidemic [18], [19]. Therefore, our surveillance system, although based on voluntary participation, seems to cover a substantial percentage of IPD isolates, at least among children. The accuracy of our system may also be demonstrated by the unchanged proportion of isolates causing different IPD through ten-year surveillance. Besides, pneumonia (68–71%) was by far the most frequent diagnosis compared to meningitis, bacteremia/sepsis and other invasive pneumococcal diseases, which also points to an accurate surveillance [20].

Our study demonstrated a significant decrease in total IPD incidence among children <2 years old (IR 68.7 to IR 29.6, p<0.001) and children 2 to 4 years (p<0.04) when pre-vaccination (2003–2007) and post-vaccination (2008–2012) periods were compared. By the end of the study period, IPD caused by serotypes in PCV7 was reduced by 95.6% and IPD caused by 6 serotypes added in PCV13 was reduced by 83.9% among this target population. Similar effects have been observed in other countries. Routine use of PCV7 in Canada and the United States, has also lead to near eradication of IPD caused by serotypes included in the formula in both children and adults [5], [21]. In 2007, the Active Bacterial Core surveillance of nearly 30 million Americans demonstrated the near-elimination of IPD caused by PCV7 serotypes in children <5 years old, and a 76% decline in total IPD within this age group. Indirect or “herd” effect among the non-vaccinated population was also reported: 94% decline in VT-PCV7 associated IPD and 45% decline in all IPD [8].

Nevertheless, concern was expressed for Latin American countries with different serotype distribution including a high proportion of isolates of non PCV7 serotypes 1 and 5 [22]. For instance, potential coverage of PCV7 in Uruguayan children ≤5 years old before vaccine introduction was only 49% but reached 76% when serotypes 1 and 5 were added [7]. National Health authorities decided to introduce PCV7 anyway, with the understanding that it would be replaced by another extended-formulation vaccine, as soon as it was commercially available.

As expected, a recently published case-control study in Uruguay has shown the benefit of PCV7 to prevent serotype-specific IPD among vaccinated population, with an effectiveness of 91.5% for >1 dose and 94.0% for >2 doses [10]. Other studies, such as variation in hospitalizations for pneumonia and meningitis at the reference pediatric hospital, and population-based analysis of consolidated pneumonia, also found significant declines in PCV7 associated IPD among vaccinated children [23], [24]. The incidence of consolidated pneumonia for children <5 years decreased from 1197 during the pre-vaccination period to 833 during 2009–2011.

This is the first nationwide report to evaluate the added effect of consecutive immunization with PCV7 and PCV13 based on laboratory records.

In 2010, PCV7 was replaced by PCV13 at the immunization schedule and a single dose catch up was offered to all children <5 years old, already vaccinated with PCV7. A subsequent and steady decline in incidence rates of the 6 serotypes added in PCV13 was observed in 2011 and 2012 for children <2 years old, and also for children 2 to 4 years old, with consistent decrease in IPD incidence caused by any serotype. A similar situation had been observed in England and Wales, where VT-PCV13 type disease decreased by half in children aged <2 years old, after PCV13 introduction in 2010 [25].

In our study, certain variations in annual IPD rates among children <2 years old were observed before PCV7 introduction (80/100,000 in 2005 and 49/100,000 in 2007), probably due to non vaccine-related effects, as secular variations in frequency of serotypes 1 and 5 related disease. Cyclical frequency variation and outbreaks in confined populations have been described for these serotypes [26].

In order to evaluate the separate effects of each vaccine, analyses of relative frequencies of serotypes included in VT-PCV7 and VT-PCV13 were compared between 3 periods. All vaccine-serotypes showed decreased frequency over time, except serotype 3, which increased both among vaccinated and non vaccinated IPD patients. To our knowledge, this situation has not been reported elsewhere. One possible explanation is that for the first time, licensure of PCV13 was based on non-inferiority trials using serological end-points. The objective was to demonstrate similar and acceptable immunogenicity profiles for additional serotypes compared with PCV7 serotypes. Interestingly, these studies showed that before toddler dose, the lowest antibody titer was recorded for serotype 3, although it reached accepted values after the booster dose [27]. In any case, our observations need to be confirmed during the next years.

In contrast with the decline in IPD observed among the vaccinated population, our surveillance showed an increase in total number of IPD cases among adults immediately after PCV7 introduction, especially among those ≥60 years of age.

The number of adult IPD isolates before infant vaccination was low, thus small improvements in surveillance might result in large percentage changes.

This increase may be interpreted as a bias as it is an unexpected result, but we could not identify any particular changes in reporting protocols and procedures. Hence, in view of the introduction of conjugate vaccines even for adults, awareness about the importance of pneumococcal surveillance may have increased among reporting institutions. However, surveillance of pneumococcal meningitis, which is mandatory and unlikely to be biased by changes in diagnostic criteria or compliance with surveillance, also showed a 45% increase in the number of isolates between pre- and post-vaccine periods for patients ≥5 years old, comparing with 59% reduction for children <2 years old.

Interestingly, after 2009, IPD incidence among adults remained stable, because decrease in vaccine serotypes was offset by increase in non vaccine serotypes. Reduced benefits of PCV7 vaccination have already been demonstrated for vaccinated children among groups with lower socioeconomic status and different distribution of IPD serotypes, such as Alaskan natives [28]. Reduced indirect effects have also been reported in Australia, where net increase of IPD was observed among adults, as a result of serotype replacement of VT- PCV7 by NVT [29].

Although in our study the net benefit of conjugate vaccines among children <2 years old was apparent, increases in NVT associated IPD were already observed. Emerging serotypes corresponded mostly to already relatively frequent serotypes, such as 12F and 24F, which increased both their frequencies and the number of isolates significantly both within vaccinated and unvaccinated population. During the pre-vaccination period, serotype 12F ranked 12^th^ among Uruguayan children <5 years old, and was more frequent than 2 PCV serotypes, 19F and 4 [7]. Thus, these results are in agreement with the hypothesis that capsular switch is not the main driving force for serotype replacement, at least in the short term [3]. The results also highlight the importance of national surveillance of IPD, as emerging serotypes are not the same in each country or region. For instance, in the USA, the only major serotype that increased its frequency from 2008 to 2011 was 35B [30].

Percentage of isolates covered by PPSV23 among patients ≥5 years old has remained more or less unchanged after conjugate vaccines introduction because most emerging NVT are included in the formula. Thus, combination of PCV13 and 23-valent PV would be an accurate approach for older children and adults at high risk for invasive pneumococcal disease. In any case, continuous high-quality surveillance to monitor long-term vaccine effects is mandatory, as it is recommended by World Health Organization [31].
